# Unprecedented large inverted repeats at the replication terminus of circular bacterial chromosomes suggest a novel mode of chromosome rescue

**DOI:** 10.1038/srep44331

**Published:** 2017-03-10

**Authors:** Hela El Kafsi, Valentin Loux, Mahendra Mariadassou, Camille Blin, Hélène Chiapello, Anne-Laure Abraham, Emmanuelle Maguin, Maarten van de Guchte

**Affiliations:** 1 Micalis Institute, INRA, AgroParisTech, Université Paris-Saclay, 78350 Jouy-en-Josas, France; 2 MaIAGE, INRA, Université Paris-Saclay, 78350 Jouy-en-Josas, France

## Abstract

The first *Lactobacillus delbrueckii* ssp. *bulgaricus* genome sequence revealed the presence of a very large inverted repeat (IR), a DNA sequence arrangement which thus far seemed inconceivable in a non-manipulated circular bacterial chromosome, at the replication terminus. This intriguing observation prompted us to investigate if similar IRs could be found in other bacteria. IRs with sizes varying from 38 to 76 kbp were found at the replication terminus of all 5 *L. delbrueckii* ssp. *bulgaricus* chromosomes analysed, but in none of 1373 other chromosomes. They represent the first naturally occurring very large IRs detected in circular bacterial genomes. A comparison of the *L. bulgaricus* replication terminus regions and the corresponding regions without IR in 5 *L. delbrueckii* ssp. *lactis* genomes leads us to propose a model for the formation and evolution of the IRs. The DNA sequence data are consistent with a novel model of chromosome rescue after premature replication termination or irreversible chromosome damage near the replication terminus, involving mechanisms analogous to those proposed in the formation of very large IRs in human cancer cells. We postulate that the *L. delbrueckii* ssp. *bulgaricus*-specific IRs in different strains derive from a single ancestral IR of at least 93 kbp.

The thermophilic lactic acid producing bacterium *Lactobacillus delbrueckii* is mainly known for its application in dairy fermentations, notably in the production of yogurt (*L. delbrueckii* ssp. *bulgaricus (L. bulgaricus*)) and cheese (ssp. *lactis* and ssp. *bulgaricus*), and for its probiotic potential^1^,^2^,^3^. The genomes of these bacteria show a number of interesting characteristics, among which a relatively high GC content at third codon positions and the presence of exceptionally high numbers of pseudogenes and incomplete metabolic pathways, indicating that they are in a state of ongoing evolution, in what appears to be an adaptation to the milk environment of an ancestor that probably evolved in the mammalian digestive tract^4^,^5^.

One very intriguing observation we made when resolving the first *L. bulgaricus* genome sequence was that of the presence of a 47.5 kbp inverted repeat (IR) in the replication terminus region^5^. Not only is the presence of such a naturally occurring very large IR *per se* unprecedented in circular bacterial genomes, where even small IRs are generally unstable^6^,^7^, but the repeated sequence also includes the putative *dif* site, the recombination site involved in the resolution of chromosome dimers^8^. Consequently, the chromosome contains two putative *dif* sites in opposite orientation instead of the unique site found in other circular bacterial chromosomes, a configuration expected to complicate the resolution of dimers that regularly form during chromosomal replication^9^ as recombination between two *dif* sites of opposite orientation (among the four *dif* sites in an *L. bulgaricus* chromosome dimer) would result in the inversion of a part of the dimer rather than its resolution^5^,^10^. These observations prompted us to investigate if similar IRs could be found in other genomes.

We present a comparative analysis of 10 *L. delbrueckii* genome sequences, representing 5 strains of the ssp. *lactis* and 5 strains of the ssp*. bulgaricus*, with a focus on the replication terminus region. We show that very large naturally occurring IRs are found in the replication terminus region of all 5 ssp. *bulgaricus* chromosomes, but in none of the ssp. *lactis* chromosomes or other fully sequenced circular bacterial chromosomes in the public databases. A comparison of the *L. bulgaricus* replication terminus regions and the highly homologous corresponding regions without IR in the ssp. *lactis* genomes leads us to propose a model for the formation and evolution of the IRs. The DNA sequence data are consistent with a novel model of chromosome rescue after premature replication termination or irreversible chromosome damage near the replication terminus, involving the formation of a very large IR through mechanisms analogous to those proposed in the formation of very large IRs in human cancer cells.

## Results

### Strain diversity is non-evenly distributed over the *L. delbrueckii* core-genome

In this study we used 4 complete and 6 nearly complete *L. delbrueckii* genome sequences, together representing 5 *L. delbrueckii* ssp. *bulgaricus* strains and 5 *L. delbrueckii* ssp. *lactis* strains. The genome sequences were aligned to determine the core-genome, consisting of genome segments (>1 kbp) shared by all 10 strains studied.

The 10 genomes are essentially colinear (results not shown) and share a common backbone of 1.17 Mbp, covering 62% of the individual completely sequenced ssp. *bulgaricus* genomes (strains ATCC11842, ATCC BAA365, 2038) and 55% of the ssp. *lactis* NDO2 genome. The 5 strains of the ssp. *lactis* share 1.38 Mbp, the 5 strains of the ssp. *bulgaricus* 1.53 Mbp. 22,703 polymorphic sites were found among the 5 ssp*. lactis* strains studied (1.65% of the ssp. *lactis* core genome), and 15,390 among the 5 ssp. *bulgaricus* strains (1.00% of the ssp. *bulgaricus* core genome) (Table 1).

**Figure 1:**
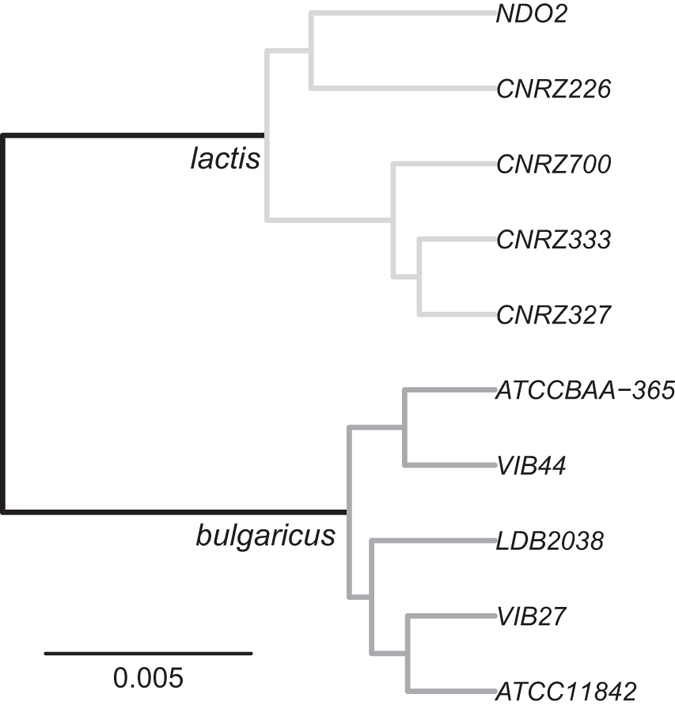
Core genome based phylogeny of *L. delbrueckii* strains. Branch length represents the expected number of substitutions per base.

The phylogeny of the strains reconstructed on the basis of the core genome shows a clear separation between the ssp. *bulgaricus* and the ssp. *lactis* clades (Fig. 1), consolidating earlier 16S rRNA and MLST-based strain classifications^4^. When *Lactobacillus acidophilus* is used as an external root, the root is placed on the branch separating the two clades (results not shown).

**Figure 2:**
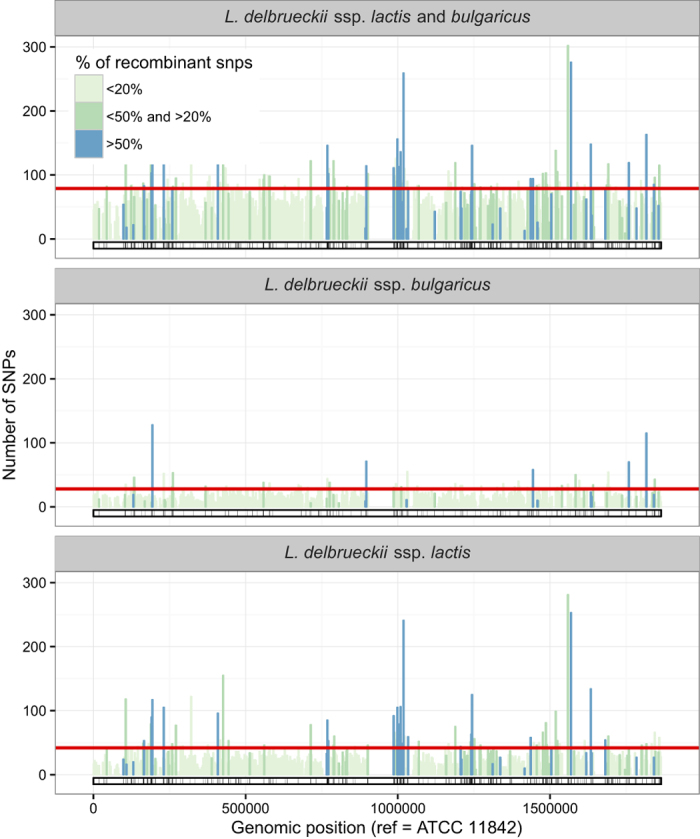
Local density of SNPs along the core-genome. SNPs are counted over non-overlapping windows of 2.5 kbp. The fraction of recombinant SNPs is computed by averaging the probabilities of being recombinant over all SNPs in the window and color-coded. The red lines indicate the SNP count thresholds: counts above the threshold indicate that the window contains more SNPs than expected by chance if the SNPs were uniformly distributed (p < 0.05 after fdr correction for multiple testing). Empty zones correspond to regions not belonging to the core genome of *L. delbrueckii*. SNP counts and fraction of recombinant SNPs are computed from the complete core genome of the 10 *L. delbrueckii* strains (top panel), of the 5 *L. delbrueckii* ssp. *bulgaricus* strains (middle panel), or of the 5 *L. delbrueckii* ssp. *lactis* strains (bottom panel). The barcode at the bottom of each panel highlights the fragments detected as recombinant (black) in at least one strain of the corresponding strain set.

A more detailed analysis of the core genome, using local density of polymorphic sites in a sliding 2.5 kbp window, shows that the diversity between the 10 strains is not evenly distributed over the core genome (Fig. 2), a feature which may be indicative of homologous recombination within the core genome during evolution. Clear examples of horizontal gene transfer and recombination outside the core genome have been described elsewhere^4^. Remarkably, in the ssp. *lactis* strains studied a relatively high density of SNPs is observed in a region (around position 1,000,000 in Fig. 2) close to the replication terminus, which itself is not included in the core genome, i.e. not conserved among strains (around position 945,000 in Fig. 2).

### Homologous recombination and mutation as driving forces of core genome diversity

In order to gain more insight in the driving forces of evolution in the core genome of the two *L. delbrueckii* subspecies, we used ClonalFrame^11^, a method developed to infer the clonal relationships of bacteria and assess the contributions of point mutations and recombination. ClonalFrame distinguishes the latter (i.e. genetic exchange) from the former assuming that recombination events introduce a constant and elevated rate of substitution in a contiguous sequence region while point mutations are scattered and rare. Under this assumption, a high local density of SNPs is the footprint of past recombination events. When applied to *L. delbrueckii*, ClonalFrame infers that similar proportions of the genome-wide observed diversity (SNPs) can be attributed to recombination events in the two subspecies studied: 40% in the ssp. *lactis* core-genome, and 36% in the ssp. *bulgaricus* core-genome (Table 1). The size of the identified recombinant tracts ranged from 10 to 1688 bp, with an average that was essentially the same among the ssp. *lactis* strains (204 bp) and among the ssp. *bulgaricus* strains (189 bp) (Table 1).

The results of this study indicate that the genomes of the two ssp. have generally conserved structures, and that recombination events contributed to comparable extents to strain diversity in the two ssp. The replication terminus region represents a major exception to this overall similarity and does not make part of the common core-genome.

### *L. delbrueckii* ssp. *bulgaricus* chromosomes, but not *L. delbrueckii* ssp. *lactis* chromosomes, contain an unprecedented very large inverted repeat in the replication terminus region

A very intriguing observation made in the replication terminus region of the first complete genome sequence of *L. delbrueckii* ssp. *bulgaricus* concerned the presence of a very large, 47.5 kbp, inverted repeat (consisting of repeats of 23 kbp each that enclose a unique central region of 1.4 kbp)^5^. In the *L. bulgaricus* ATCC11842 genome, this IR is situated at positions 918952–966484, which coincides with a region that is not covered by the core genome of the 10 strains in the present study (Fig. 2), indicating that important differences between strains exist in this region. We therefore subjected the replication terminus regions of the genomes to closer examination.

**Figure 3:**
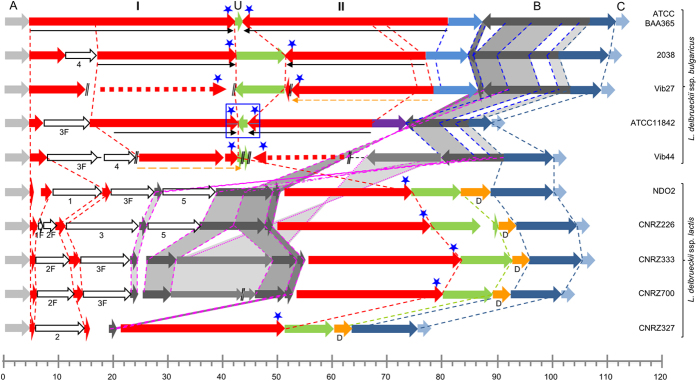
Schematic representation of replication terminus regions of 10 *L. delbrueckii* genomes. (**A**) “anchor” sequence, identical in all genomes; I, left-hand repeat; U, unique central sequence; II, right-hand repeat (in strain ATCC BAA365). Parts of I and II that are underlined with black arrows constitute the IR in the respective ssp. *bulgaricus* strains. Thin-lined orange arrows indicate duplicated regions (sequencing depth two times higher than adjacent regions) in strains Vib27 and Vib44 (cf Fig. 5). Dotted red arrows indicate probable positions of the duplicate regions. The blue rectangle indicates the approximate position of the replication terminus in the ATCC 11842 genome^5^. Blue stars indicate positions of putative *dif* sites. //, separation between two sequence scaffolds. Region (**B**) is translocated to the opposite replichore in the ssp. *lactis* compared to the ssp. *bulgaricus*. Identical colors indicate homology between strains. 1, 2, 3, 4, and 5 indicate additional regions of homology, where (F) indicates that only a part (Fragment) of the sequence is present. Scale, size (kbp).

Our analysis of the earlier published genome sequences of *L. delbrueckii* ssp. *bulgaricus* strains ATCC BAA365 and 2038 revealed the presence of large IRs resembling the one in the ATCC11842 genome, with sizes of 76.3 kbp and 59.8 kbp, respectively (37.6 kbp and 25.5 kbp, respectively, for the repeated sequence, and 1 and 8.8 kbp for the central region between the repeats) (Fig. 3, Table 2).

**Figure 4:**
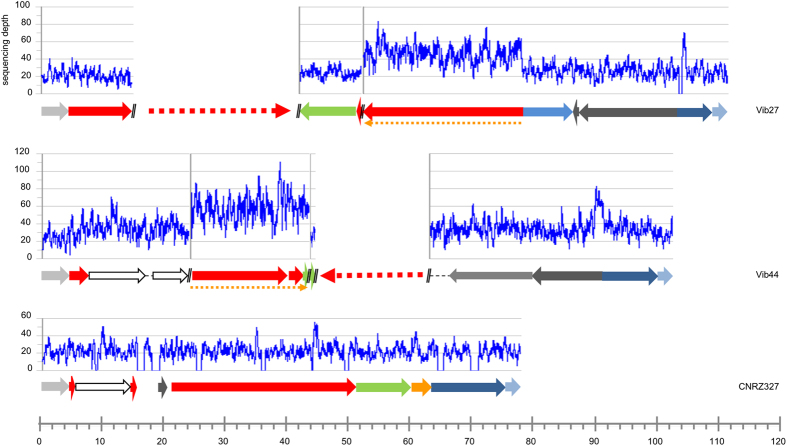
Sequencing depth in the vicinity of the replication terminus of the Vib27, Vib44 and CNRZ327 genomes. Schematic representation of the replication terminus regions of *L. delbrueckii* ssp. *bulgaricus* strains Vib27 and Vib44 and *L. delbrueckii* ssp. *lactis* strain CNRZ327; see Fig. 3 for details. //, separation between two sequence scaffolds. Graphs represent sequencing depth after whole genome sequencing (454 paired-end sequencing, Roche Life Sciences). Orange dotted lines indicate regions where sequencing depth is twice as high as in surrounding regions, indicating that these regions are repeated in the respective chromosomes. Horizontal scale, size (kbp).

Several lines of evidence indicate the presence of large IRs in the *L. delbrueckii* ssp. *bulgaricus* strains Vib27 and Vib44 too, even if the recently established genome sequences are not complete: 1) sequence assembly ends (i.e. reaches the end of a contig and a scaffold) near the replication terminus, as expected when a large repeated sequence is present at this site (only one copy of the repeated sequence is expected to be assembled); 2) the average sequencing depth of a 25.1 kbp (strain Vib27) or 18.7 kbp sequence (strain Vib44) appears to be twice as high as in the adjacent regions, as expected if this sequence is duplicated in the genome (Fig. 4); 3) these duplicated regions coincide with the duplicated regions (inverted repeats) in the fully sequenced genomes of *L. delbrueckii* ssp. *bulgaricus* strains ATCC11842, ATCC BAA365, and 2038 (Fig. 3). The size of the unique sequence between the repeats could not be precisely determined in the nearly complete genome sequences but appears to be at least 8.8 kbp in strain Vib27 and 1.3 kbp in strain Vib44, based on homology of contigs of normal sequencing depth with the central non-duplicated region of the IR in strain 2038 (Fig. 3). The complete IRs in strains Vib27 and Vib44 thus have sizes of at least 59 and 38.7 kbp, respectively.

Remarkably, none of the 4 newly sequenced ssp. *lactis* genomes shows any sign of the presence of a large IR in the replication terminus region, nor does the earlier published complete genome sequence of *L. delbruecki* ssp. *lactis* NDO2. The presence of a large IR in the replication terminus region of the chromosome thus appears to be a distinguishing characteristic of the ssp. *bulgaricus*.

In other bacterial genomes, inverted repeats of this size have to our knowledge only been reported in certain, normally linear, *Streptomyces* chromosomes that had circularized after UV-induced telomere deletion^12^, and in an artificially modified *Escherichia coli* chromosome where the formation of a large IR was observed in an *sbcCD RecA* background only^13^. In order to see if similar genome structures which may not have been reported in literature could be detected in other bacteria, we analyzed 1368 completely sequenced circular bacterial genomes present in the public databases, including those of several lactobacilli from the acidophilus group to which *L. delbrueckii* belongs^14^. We identified 5 genomes containing IRs with repeats >8 kbp separated by a unique central region <10 kbp. For only 3 of these genomes the IR was situated in the replication terminus region, namely the 3 earlier published *L. delbrueckii* ssp. *bulgaricus* genomes mentioned above (strains ATCC11842, ATCC BAA365, and 2038), with sizes varying between strains from 23 to 37.6 kbp for the repeated sequence and from 1 to 8.8 kbp for the central region between the repeats. Much smaller IRs, containing repeated sequences of 8.7 or 9.4 kbp and not situated in the replication terminus region, were detected in the genomes of *Streptococcus suis* strain D9 and *Magnetospirillum magneticum* strain AMB-1, respectively. Among natural completely sequenced circular genomes, a very large size IR in the replication terminus region like the one originally identified in strain ATCC11842 thus appears to be an exclusive property of the *L. delbrueckii* ssp. *bulgaricus* genome.

### Important size variation of the conserved IR foretells its disappearance

A more detailed analysis of the replication terminus regions in the three completely sequenced *L. delbruecki* ssp. *bulgaricus* genomes (ATCC11842, ATCC BAA365, and 2038) reveals that the three IRs, although differently sized (Fig. 3), are largely homologous between strains (>97% sequence identity in the conserved parts of the IR). The deduced structures for the nearly complete sequences of strains Vib27 and Vib44 follow the same pattern.

The repeat size varies from 18.7 kbp for strain Vib44 to 37.7 kbp for strain ATCC BAA365. Of interest, while the beginning of the left-hand repeat of strain ATCC BAA365 (i.e. the left-hand part of sequence I in Fig. 3) is conserved in all 5 ssp. *bulgaricus* strains, the beginning of the right-hand repeat of this strain (i.e. the right-hand part of sequence II in Fig. 3) is not present in the other strains. Similarly, the beginning of the right-hand repeat of strains 2038 and Vib27 is not present in strains ATCC11842 or Vib44. In some *L. bulgaricus* strains, relatively large additional sequences are present within the region that is homologous to the ATCC BAA365 repeat I sequence, but just outside of the IR in the strains concerned (Fig. 3, sequences 3F and/or 4). The size of the central sequence in between the repeats varies from 1 to 8.8 kbp (Fig. 3, sequence U), where the smaller sequences appear to be derived from the largest sequence present in strains 2038 and Vib27.

**Figure 5:**
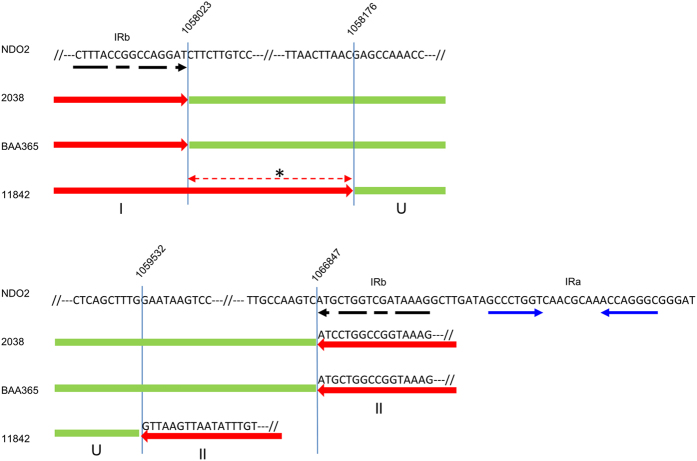
Sequence detail of borders between IR repeats and central sequences. Borders between repeats and central sequences in the *L. delbrueckii* ssp. *bulgaricus* strains 2038, ATCC BAA365 and ATCC11842 are projected on the sequence of the *L. delbrueckii* ssp. *lactis* strain NDO2 (which contains no large IR). The ssp. *bulgaricus* sequences are identical to the corresponding ssp. *lactis* sequences unless indicated otherwise. Blue arrows, small IR (IRa) in strain NDO2; broken black arrows, imperfect inverted repeat (IRb) in strain NDO2; I, II, and U as in Fig. 3; *region making part of the repeat in strain ATCC11842, but making part of the non-repeated central sequence in strains ATCC BAA365 and 2038. The presented NDO2 sequences are representative for all 5 ssp. *lactis* strains in this study.

At first sight, the simplest hypothesis to explain these observations seems to assume that an ancestral IR consisted of repeats resembling those present in strain ATCC BAA365 (the largest repeats in our dataset) of which sequences 3 and 4 may have made part, and the largest central sequence present in strains 2038 and Vib27, and that all present IRs have derived from this ancestral repeat by differential deletions. However, when taking a closer look it appears that a small part of the repeat sequence in strain ATCC11842 (Fig. 5, region indicated by *) is not repeated in strain ATCC BAA365, but in the latter strain makes part of the non-repeated central sequence (U in Fig. 3). The IR in strain ATCC11842 can therefore not be a simple deletion derivative of an IR resembling the one in strain ATCC BAA365. If the IRs derive from a common ancestral repeat, this repeat must have had a different structure (where the region indicated by * in Fig. 5 makes part of the repeated sequence; see Discussion).

Under the hypothesis of a common ancestral IR (see Discussion), the size differences between the highly homologous sequences in the central region and in the repeats indicate that in *L. bulgaricus* the IR is in the course of elimination. Independent of the hypothesis of a common ancestor this conclusion is supported by the earlier observation made in *L. delbrueckii* ssp. *bulgaricus* ATCC11842 that many genes in the IR are fragmented pseudogenes^5^ (see Supplementary Table S1).

### Comparison of subspecies provides indications on divergent subspecies evolution and a possible mechanism of IR formation

While in the absence of sequence information from the *L. delbrueckii* ancestor it is difficult to retrace evolution history, we compared the genomes of the present-day subspecies in search of indications that might inform us on the possible mechanism of IR formation and evolution. In the ssp. *lactis* strains, a non-repeated region with a high degree of identity to the ssp. *bulgaricus* repeat is found, of which the beginning and end correspond to the beginning and the end of the left-hand repeat of strain ATCC BAA365 (Fig. 3, sequence I) and are conserved in all *L. delbrueckii* strains examined, ssp. *bulgaricus* and ssp. *lactis* alike. Within these ssp. *lactis* regions, several parts are found that are not found in the ssp. *bulgaricus* genomes (Fig. 3, sequences 1, 2, 5), and also parts of a sequence that is exterior to the repeats, situated to the right of the IRs in Fig. 3, in the ssp. *bulgaricus* strains (sequence B). In the two subspecies, the latter sequences are present in opposite orientations and situated at opposite sides of the replication terminus. This observation can be explained by an inversion around the replication terminus, a type of genome rearrangement regularly observed in circular bacterial genomes^15^,^16^, distinguishing the two *L. delbrueckii* subspecies.

The ssp*. lactis* strains all contain the longest version of the sequence that separates the inverted repeats in the ssp. *bulgaricus* strains (8.8 kbp; Fig. 3, sequence U). In the ssp. *lactis*, this sequence is flanked by two sequences of 16 bp that form an imperfect IR (Fig. 5, IRb), where the 16 bp sequence to the left of region U is identical to the extremity of the repeated sequence I (in red in Figs 3 and 5) in the ssp. *bulgaricus* strains 2038 and ATCC BAA365. Furthermore, in the ssp. *lactis*, region U and its 16 bp flanking sequences are immediately followed by a 3.8 kbp region (D in Fig. 3) which is absent from the ssp. *bulgaricus* genomes. Within this region and in close proximity to region U, a short IR is present (Fig. 5, IRa)). These features provide important indications on the possible mechanism of IR formation in the ssp. *bulgaricus* (see Discussion).

Analogous to the hypothesis put forward for the ssp. *bulgaricus* genomes, and taking the status of sequence B in the two subspecies into account, we speculate that a common ancestor of the ssp*. lactis* strains contained sequence I (Fig. 3) as well as the sequences U, 1, 2, 3, 5, and B, and that the ssp*. lactis* strains studied here are deletion derivatives of this strain.

Together, the observations in the two *L. delbrueckii* subspecies indicate that extensive recombination, translocation, inverted duplication and deletion formation have taken place in the replication terminus region of the genomes and produced a hallmark difference between the ssp. *lactis* and *bulgaricus,* where the latter contains a very large IR that is absent from the former.

## Discussion

The genomes of the *L. delbrueckii* subspecies *bulgaricus* and *lactis* are in an active phase of evolution, as witnessed by an aberrant GC content at third codon positions^5^ and remarkably high numbers of pseudogenes in both subspecies^4^,^5^. In the present study we highlight a new aspect of genome evolution in these bacteria, focusing on the replication terminus region which turns out to be highly variable, between and within the two subspecies.

We show that very large IRs are present in this region in all ssp. *bulgaricus* genomes studied, but none of the ssp*. lactis* genomes. Moreover, from an analysis of 1368 completely sequenced circular bacterial genomes it appears that IRs of the size described in this study are unique to *L. delbrueckii* ssp. *bulgaricus*, suggesting that the formation of such a structure is an extremely rare event, that other bacteria have more efficient ways to remove a very large IR when formed, or that very large IRs generally interfere with viability or fitness and thus rapidly disappear from a population. More efficient removal of very large IRs in other bacteria does not seem a very likely explanation, however. While it is known that, at least *in vitro*, small IRs can be entirely excised during DNA replication through slippage of the DNA polymerase on small direct repeats at the basis of the stem-and-loop structure formed by the IR^17^, this mechanism is not expected to remove very large inverted repeats like those present in the *L. delbrueckii* ssp. *bulgaricus* genomes as they will probably never form a stem-and-loop structure *in vivo*. Furthermore, the complete removal of a very large IR may not be desirable or possible as the IR may carry important or essential genes or other functions, like the *dif* site in the replication terminus region in the present study. Homologous recombination between the repeated sequences, a process that can efficiently eliminate tandem repeats and the sequence that separates them to leave only one copy of the repeated sequence, would only inverse the central region of the IR. Gradual deletion involving other mechanisms thus appears to be the only possibility to clear the genome of a large IR. As a consequence, very large IRs are expected to be relatively persistent once formed, and the rarity of very large IRs in bacterial genomes therefore seems to indicate that they rarely form or are generally not viable. These considerations and the strong resemblance of the different IRs studied here, including the same chromosomal localization in the different strains, lead us to postulate that the large IRs in the ssp. *bulgaricus* genomes most likely derive from a common ancestral IR, rather than being the result of independent IR-generating events.

Our observations in the present-day ssp. *bulgaricus* and *lactis* strains lead us to propose a model to account for the formation of a large ancestral IR in *L. bulgaricus* and its subsequent evolution. The origin of large IRs has been studied in human cancer cells, where they have been implicated in extensive gene amplification, and in yeast. In both organisms, double-strand DNA breaks (DSBs) near to short IR sequences appear to be at the origin of the formation of such structures^18^,^19^,^20^. The free 3′ end of one of the broken DNA strands is thought to fold back on an inverted repeated sequence (which may be as short as 4 bases^21^) in the near vicinity (10 to 30 bp away), and serve to prime DNA synthesis of the complementary strand, eventually resulting in the formation of a large inverted repeat. This mechanism has also been evoked to explain the formation of a large IR in *E. coli*, albeit starting from a much larger, artificially introduced, initial inverted repeat and only observed in an *sbcCD recA* background^13^, and in the formation of an IR in the linear chromosome of *Streptomyces griseus* after UV-induced telomere deletion^12^. At a difference with these cases, where the repeats are practically head to head, the repeated sequences in *L. delbrueckii* ssp. *bulgaricus* are separated by 1 to 8.8 kbp of unique sequence, however. The 8.8 kbp region is also present in the ssp. *lactis* strains, where it is flanked by a 16 bp imperfect inverted repeat (Fig. 5, IRb) and nearly immediately followed by a small IR (Fig. 5, IRa).

**Figure 6:**
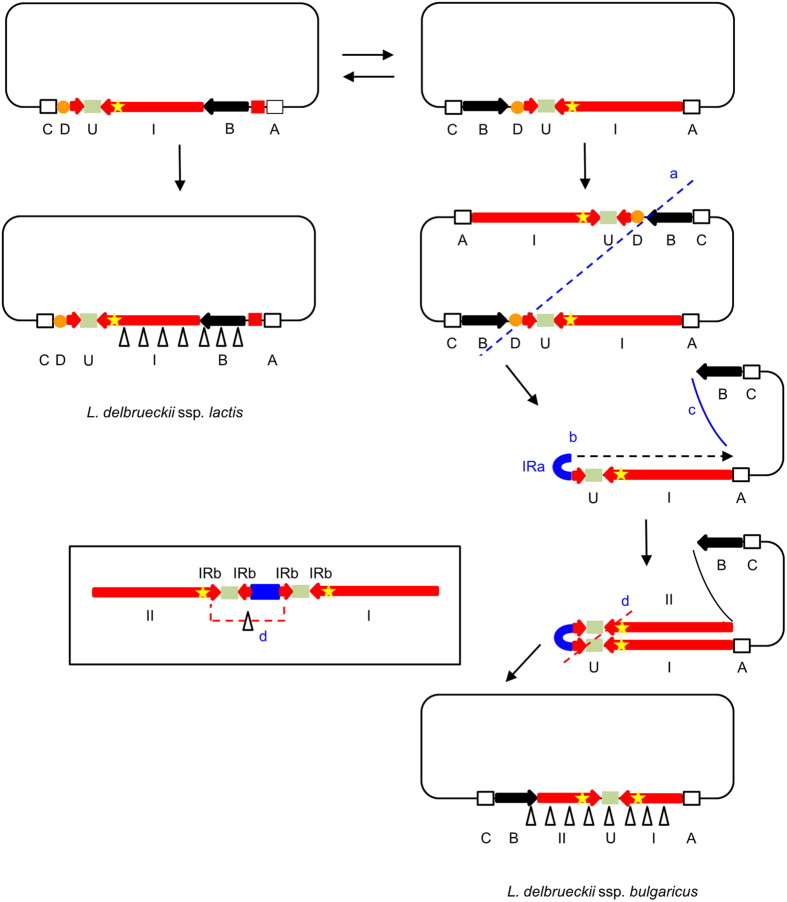
Putative model of divergent evolution in *L. delbruecki* and generation of a very large IR near the replication terminus in the subspecies *bulgaricus*. Schematic representation of common ancestor (top, two alternative structures differing in the position of region B), ssp. *lactis* (bottom left), ssp. *bulgaricus* (bottom right) and intermediary chromosomes. Regions A, I, II, U, D, B, C as in Fig. 3. IRa (blue rectangles) and IRb (pairs of red arrowheads), small inverted repeats (cf Fig. 5); yellow stars, *dif* sites. Left, evolution of ssp. *lactis* via multiple deletions (Δ) and/or insertions. Right, evolution of ssp. *bulgaricus*: a, septum closure isolates a chromosome which lacks region D. The picture represents the cleavage of a chromosome dimer at septum closure as one of two alternatives to generate an incomplete chromosome discussed in the text. In this process, tens of kbp of DNA sequence to the left of the hatched line may be degraded^26^. Rescue of the chromosome lacking D, to the right of the hatched line, proceeds via generation of an ancestral IR (b) and DSB repair (c). d, homologous recombination between direct repeats (see insert) deletes part of one copy of the repeat sequence, leaving a long non-repeated central sequence in the final IR. Multiple deletions and/or insertions gave rise to the present-day chromosomes. Figures are not drawn to scale.

We propose a model in which a DSB occurred in the vicinity of this small IR (IRa) in the replication terminus region of an ancestral genome that resembled an intermediate between the present ssp. *lactis* and ssp. *bulgaricus* genomes, containing the order of sequences A, I, U, D, B, C (Figs 3 and 6). In the replication terminus region, DSBs may be provoked by stalling replication forks (and are generally repaired by recombination processes involving RecBCD and usually also RecA to restore a normal chromosome^22^). Alternatively, DSBs in this region may result from the cleavage (as opposed to resolution via *dif*) of chromosome dimers that regularly form during the replication of circular chromosomes as a consequence of recombination between sister chromatids^9^, at septum closure^23^,^24^,^25^,^26^. We hypothesize that either the one or the other left one of the daughter cells with an incomplete chromosome, resembling a ssp. *lactis* chromosome in Fig. 3 (apart from the position of sequence B) disrupted at the right side of sequence U, making the restoration of a complete normal chromosome by homologous recombination impossible (Fig. 6). Chromosome rescue would then have passed through a phase of fold-back synthesis primed by intra-strand self-annealing of the short IR situated to the right of sequence U (Fig. 5, IRa), and subsequent DSB repair resulting in the formation of an ancestral IR (Fig. 6). Consistent with this hypothesis, a 3.8 kbp region (D in Figs 3 and 6) directly adjacent to the fore mentioned region U in the ssp. *lactis* genomes is lacking in the ssp. *bulgaricus* genomes.

It is worth mentioning that the ancestral very large IR would have had a spacer (central non-duplicated region) of only 8 bp, comparable to those observed in eukaryotic very large IRs, and a total length of at least 93 kbp (2 times the length of region I (Fig. 3) in strain ATCC BAA365 plus 2 times the length of region U (Figs 3 and 5) in strains 2038 and NDO2). The IRs with their long spacers in the ssp. *bulgaricus* strains studied would have been derived from this ancestral IR by deletions involving short repeats, like the imperfect repeat (IRb) depicted in Fig. 5, present in the ancestral genome before generation of the large IR. These short IRs in the ancestral genome appear also as short direct repeats (DRs) after generation of the large IR (Fig. 6, bottom insert). Deletion of the region between these DRs would eliminate a part of one copy of the ancestral repeat region and thus explain why in the present genomes the repeated sequences are separated by a long non-repeated central sequence (Fig. 6). The feasibility of deletion of very long sequence regions between remote short DRs has been described earlier^27^,^28^.

The sequences at the border of repeats and central sequence of the IRs in strains 2038 and ATCC BAA365 are fully explained by this model (Fig. 5). For strain ATCC11842 the ancestral small IR (equivalent to IRb in Fig. 5) that would have been involved could not be identified in the present-day ssp. *lactis* strains, probably because additional sequence modifications have taken place since, in the ssp. *bulgaricus* or the ssp. *lactis* lineage, that prohibit reconstruction of the evolution events. Additional sequence modifications in either or both subspecies, among which the translocation of sequence B (Fig. 3) and deletions at the right side (Fig. 3) of the IR in *L. bulgaricus*, are probably also the reason why we cannot identify the point where, after IR generation, DSB repair has taken place to join the end of the newly formed repeat and the other free end of the broken chromosome (step c in Fig. 6). This process may have involved illegitimate recombination (non-homologous end-joining), although in that case it is not clear why IR formation occurred in the first place, instead of direct joining of the broken ends. Another possibility is that fold-back DNA synthesis served to generate homologous ends at either side of the break to allow repair by homologous recombination, the principal route of DSB repair in bacteria^29^. The abundant IS elements in the *L. delbrueckii* genome^4^,^5^ may have played a role in this process, although no traces of their involvement could be found (anymore) in the present genomes.

Apart from these major duplication and deletion events that sculpted the general aspect of very large IRs with central non-repeated regions of up to 8.8 kbp, many smaller deletions appear to gradually eliminate the IRs. This erosion is also visible at the genetic level, where many genes in the IR appear to be pseudogenes (fragmented genes in the course of elimination through deletion formation)^5^.

The fact that, in spite of the expected rarity of the events that we hypothesize to be at the basis of very large IR formation, such IRs are consistently found in *L. delbrueckii* ssp. *bulgaricus* and, moreover, strongly resemble each other and share their localization on the chromosome, points to a unique event in the evolution of *L. delbrueckii* ssp. *bulgaricus*, shaping an ancestral IR from which the present IRs derive. This ancestral IR may have been fixed in the population through positive selection if, at the time of formation, the increased gene dosage of genes in the repeat represented an advantage outweighing the possible inconvenience of a large IR. While the repeat comprises many genes of unknown function that may have played a role in positive selection, one more tangible possibility is that duplication of the genes encoding a multidrug efflux protein and its regulator (see Supplementary Table S1, ldb1074/1075) provided an enhanced capacity to extrude toxic substances entering from the environment. Alternatively, even if slightly disadvantageous, the IR may have been fixed by genetic drift if the population went through a population bottleneck^30^,^31^. This could be a plausible explanation in the context of the domestication history of this bacterial species. As discussed above, once fixed in the population, IRs are difficult to remove.

The observations described in this study represent the first detection of naturally occurring very large IRs in circular bacterial genomes. Although we can only speculate on the events that led to the formation of these IRs in *L. bulgaricus*, the DNA sequence data suggest an analogy to the formation of large IRs described in human cancer cells, placed in a context of bacterial chromosome rescue. The fixation of these extremely rare very large IRs in the *L. delbrueckii* ssp. *bulgaricus* genome thus for the first time permits to propose a novel chromosome rescue model, alternative to the well-established existing models^22^ that are based on much more readily observable DNA sequence rearrangements.

## Methods

### Genome sequences

The genomes of 2 *L. delbrueckii* ssp. *bulgaricus strains* (Vib27 and Vib44) and 4 ssp. *lactis* strains (CNRZ327, CNRZ333, CNRZ226, and CNRZ700), were recently sequenced to near completion as described elsewhere^4^,^32^. The earlier published complete genome sequences of the ssp. *bulgaricus* strains ATCC11842^5^, ATCC BAA365^33^, and 2038^34^, and the ssp. *lactis* strain NDO2 (originally classified as belonging to the ssp. *bulgaricus*^35^, but in fact belonging to the ssp. *lactis*^4^) were retrieved from NCBI.

### Core-genomes

Complete genomes retrieved from NCBI and scaffolds from newly sequenced genomes were aligned using MOSAIC with default parameters^36^,^37^ to determine core-genomes, containing DNA sequence regions (>1 kb, including coding and non-coding sequences) shared by all *L. delbrueckii* ssp. *bulgaricus* strains, by all *L. delbrueckii* ssp. *lactis* strains, or by all strains of the two ssp.

### Phylogenetic reconstruction

Strain phylogenetic trees were computed on the basis of core genome alignments using two reconstruction methods that gave the same topology. PhyML 3.0^38^ was used to reconstruct maximum likelihood trees (substitution model GTR+I+G selected using modelgenerator^39^, 100 bootstrap replications). UPGMA trees were reconstructed using the PHYLIP package (http://evolution.genetics.washington.edu/phylip.html). Distance matrices were computed with DNAdist (Jukes-Cantor distance) after which Neighbor was used to construct the UPGMA tree (100 bootstrap replications). FigTree 1.3.1 (http://tree.bio.ed.ac.uk/software/figtree) and the R package ape^40^ were used to visualize phylogenies.

### Determination of substitution and recombination rates within subspecies

Nucleotide substitution and recombination rates within each ssp. were determined using ClonalFrame^11^, in the core-genome sequences (as defined above). Since both the UPGMA and maximum likelihood tree had a congruent topology with high bootstrap values, we fixed the maximum likelihood tree as the starting tree and allowed ClonalFrame to update only the branch lengths, not the topology. The mutation rate θ was fixed to the value given by the Watterson^41^ estimate 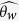
_._ In the presence of recombination, 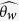 overestimates the mutation rate, which should lead to a conservative calling of recombination events. All other parameters were estimated by ClonalFrame. Three independent runs with different priors were run for 150,000 generations and samples were recorded every 100 generations after a burn-in of 50,000 generations. All runs converged to the same posterior distributions as evidenced by visual inspection of the posterior distributions and more objectively by high Effective Sample Sizes (ESS, >220 for all parameters) and low potential scale reduction factors^42^ (<1.12 for all parameters in all runs).

### Identification of large IRs in replication terminus regions of completely sequenced genomes

1368 complete circular bacterial genome sequences were retrieved from Genbank and analysed for the presence of large inverted repeats comparable to those found in the *L. delbrueckii* ssp. *bulgaricus* ATCC11842 genome^5^ using Repseek^43^ with the following parameters: -L 1000 (repetition >1000 bp), -c (circular chromosome), -i (inverted repeats). The results were filtered to keep only inverted repeats where the size of the repeated sequence was ≥8 kbp and the distance between the two copies of the repeated sequence <10 kbp, and where the two repeats were ≥98% identical. For the genomes where a large IR meeting these criteria was identified, its position with respect to the replication terminus was determined. The origin of replication was assumed to be situated near the position of the *dnaA* gene as is the case in most circular bacterial genomes^44^. A 200 kbp region at the opposite side of the chromosome was analyzed to verify the occurrence of a change of sign in the GC-skew characteristic for the replication terminus^45^. Inverted repeats in the replication terminus region were inspected manually.

## Additional Information

**How to cite this article**: El Kafsi, H. *et al*. Unprecedented large inverted repeats at the replication terminus of circular bacterial chromosomes suggest a novel mode of chromosome rescue. *Sci. Rep.* **7**, 44331; doi: 10.1038/srep44331 (2017).

**Publisher's note:** Springer Nature remains neutral with regard to jurisdictional claims in published maps and institutional affiliations.

## Supplementary Material

Supplementary Information

## Figures and Tables

**Table 1:** *L. delbrueckii* core genomes.

	Core genomes		
	10 strains ssp. *lactis* and ssp. *bulgaricus*	5 strains ssp. *lactis*	5 strains ssp. *bulgaricus*
Core genome length (bp)	1,169,615	1,377,054	1,532,043
Number of core contigs	322	308	210
Polymorphic sites	34,720 (3.00%)	22,703 (1.65%)	15,390 (1.00%)
Recombination events*	559	419	350
r/m*	0.537 (35%)	0.679 (40%)	0.563 (36%)
Tract size (bp) (median; mean)*	203; 291	141; 204	114; 189

r/m, ratio of the number of polymorphic sites resulting from recombination and the number of polymorphic sites resulting from point mutations, percentages indicate the fraction of observed diversity that can be attributed to recombination; tract size, length of recombined sequences; *estimated using Clonal Frame.

**Table 2:** Details of the replication terminus region in *L. delbrueckii* genomes.

	*ssp. bulgaricus*					*ssp. lactis*				
	ATCC BAA365	2038	Vib27	ATCC 11842	VIB44	NDO2	CNRZ226	CNRZ333	CNRZ700	CNRZ327
Complete IR size (kbp) (repeats and unique central sequence)	76.3	59.8	59	47.5	39*	NA	NA	NA	NA	NA
Size (kbp) of region between end of sequence A and start of sequence U (Fig. 3)	37.7	37.8	36	37.9	38	69.9	73	79	75	46
Size (kbp) of region between anchoring sequences A and C (Fig. 3)	107.0	105.5	101	84.1	95*	95.3	100	101	97	71

^*^Estimated size including the unassembled repeat of the IR.
