# Supplementary material for: Unprecedented large inverted repeats at the replication terminus of circular bacterial chromosomes suggest a novel mode of chromosome rescue
Source: Sci Rep. 2017 Mar 10;7:44331. doi: 10.1038/srep44331 (PMC5345009; doi:10.1038/srep44331)
Supplement: Supplementary Information [file srep44331-s1.pdf]

Unprecedented large inverted repeats at the replication terminus of circular bacterial chromosomes suggest a novel mode of chromosome rescue.

Hela El Kafsi, Valentin Loux, Mahendra Mariadassou, Camille Blin, Hélène Chiapello, Anne-Laure Abraham, Emmanuelle Maguin, and Maarten van de Guchte.

Supplementary information.

Supplementary Table S1.

Table S1. Functions encoded in the IR of *L. delbrueckii* ssp. *bulgaricus* ATCC11842.

Locus tag	Function
ldb1064	Conserved hypothetical protein (fragment)
ldb1065	Conserved hypothetical protein (fragment)
ldb1066	ABC transporter, ATP-binding protein (fragment)
ldb1067	Carbamoyl-phosphate synthase, large chain (fragment1)
ldb1068	Carbamoyl-phosphate synthase, large chain (fragment2)
ldb1069	Carbamoyl-phosphate synthase, large chain (fragment3)
ldb1070	Carbamoyl-phosphate synthase small chain
ldb1071	Conserved hypothetical protein (fragment1)
ldb1072	Conserved hypothetical protein (fragment2)
ldb1073	Conserved hypothetical protein (fragment3)
ldb1074	Putative multidrug efflux protein
ldb1075	Putative transcriptional regulator of multidrug efflux pump gene (MerR family)
ldb1076	Hypothetical protein
ldb1077	Putative transcriptional regulator (fragment1)
ldb1078	Putative transcriptional regulator (fragment2)
ldb1079	Putative integrase/recombinase
ldb1080	Putative acetyltransferase (fragment)
ldb1081	Putative ABC transporter, permease protein (fragment1)
ldb1082	Putative ABC transporter, permease protein (fragment2)
ldb1083	Hypothetical protein
ldb1084	Hypothetical membrane protein
ldb1085	Hypothetical protein
ldb1086	Putative phosphotyrosine protein phosphatase
ldb1087	Hypothetical protein
ldb1088	ABC transporter, ATP-binding protein
ldb1089	ABC transporter, permease protein
ldb1090	Hypothetical protein
ldb1091	Hypothetical protein
ldb1092	Putative transcriptional regulator (fragment)
ldb1093	Putative transcriptional regulator (fragment)
ldb1094	Putative transcriptional regulator (fragment)
ldb1095	Putative fumarate reductase (flavoprotein)
ldb1096	Putative phage protein (fragment)
ldb1097	Putative phage protein (fragment)
ldb1098	Conserved hypothetical protein (fragment)
ldb1099	Hypothetical protein
ldb1100	Hypothetical protein
ldb1101	Putative acetyltransferase
ldb1102	Putative surface layer protein (fragment)
ldb1103	Aromatic amino acid aminotransferase (fragment)
ldb1104	Peptidyl-prolyl cis-trans isomerase

Gene annotations are taken from the GenBank file. In the annotation of the ATCC11842 genome, particular attention was given to the annotation of pseudogenes (fragmented genes)[5].
